# Small sips, big losses: measuring the feeding threshold of *Lygus* (Hemiptera: Miridae) on faba bean (*Vicia faba*) seeds

**DOI:** 10.1093/jee/toag071

**Published:** 2026-04-01

**Authors:** Teresa Aguiar-Cordero, Sean Michael Prager

**Affiliations:** Department of Plant Sciences, University of Saskatchewan, Saskatoon, SK, Canada; Department of Plant Sciences, University of Saskatchewan, Saskatoon, SK, Canada

**Keywords:** faba bean, *Lygus*, electrical penetration graph, feeding behavior, seed damage, haba, Lygus, grafica de penetración eléctrica, comportamiento alimenticio, daño en semilla

## Abstract

Faba bean (*Vicia faba* L.) is an increasingly important pulse crop in Western Canada, but its production is threatened by *Lygus* spp. (Hemiptera: Miridae), which cause pod and seed damage through piercing–sucking feeding. This study examined the relationship between *Lygus* feeding duration, insect density, and the extent of scarring damage in faba bean seeds using complementary no-choice and electrical penetration graph (EPG) experiments. In no-choice assays, single adults of *L. lineolaris* and *L. elisus* confined to plants for 1, 3, 24, or 48 h produced significantly increasing damage over time (*F* = 6.24; *P* < 0.001), reaching the Canadian Grain Commission’s 1% downgrading threshold after only 3 h of feeding. Species did not differ in damage potential. EPG recordings of *L. lineolaris* feeding revealed 3 main probing waveforms: cell rupture (CR), transition (T), and ingestion (I), with nymphs spending longer in CR and I phases than adults, although waveform type, not life stage, significantly affected feeding duration. McBryde staining detected salivary deposits in a limited number of pods and seeds, primarily from males, suggesting differences in salivation behavior among life stages. Collectively, these findings demonstrate that brief feeding periods by *Lygus* can cause economically significant damage to faba bean seeds, highlighting the importance of monitoring pest activity and minimizing feeding duration to reduce quality losses. The integration of EPG behavioral data with seed damage assessments provides a basis for establishing feeding-time thresholds to guide faba bean pest management.

## Resumen

El haba (*Vicia faba* L.) es un cultivo de leguminosa cada vez más importante en el oeste de Canadá, pero su producción se ve amenazada por especies de *Lygus* (Hemiptera: Miridae), que provocan daños en vainas y semillas mediante su alimentación de tipo picador–chupador. Este estudio evaluó la relación entre la duración de la alimentación, la densidad de insectos y el grado de daño en las semillas de haba, mediante bioensayos sin elección y registros de penetración eléctrica (EPG). En los bioensayos sin elección, adultos individuales de *L. lineolaris* y *L. elisus* confinados en plantas durante 1, 3, 24, o 48 horas causaron un incremento significativo del daño con el tiempo, alcanzando el umbral del 1% de degradación establecido por la Comisión Canadiense de Granos tras solo 3 horas de alimentación. No se observaron diferencias significativas entre especies. Los registros EPG de *L. lineolaris* identificaron tres principales formas de onda de alimentación: ruptura celular (CR), transición (T) e ingestión (I), con ninfas que pasaron más tiempo en CR e I que los adultos, aunque el tipo de forma de onda, y no la etapa de vida, tuvo un efecto significativo (*F* = 1,598.35; *P* < 0.0001). La tinción de McBryde detectó depósitos de saliva en pocas vainas y semillas, principalmente de machos, lo que sugiere diferencias en el comportamiento de salivación entre etapas de desarrollo. En conjunto, estos resultados demuestran que periodos breves de alimentación por *Lygus* pueden causar daños económicos significativos en las semillas de haba, subrayando la importancia de monitorear su actividad y reducir la duración de la alimentación para minimizar pérdidas de calidad.

## Introduction

Faba bean (*Vicia faba* L.) represents an important pulse crop in Western Canada due to its strong nitrogen-fixing ability and high nutritional value (Mwanamwenge et al. 1998). In 2021, the Prairie provinces accounted for 50.5% of Canada’s total faba bean area, encompassing ∼24,807.63 hectares (Government of Saskatchewan 2023). Despite its agronomic and nutritional benefits, faba bean production is increasingly constrained by arthropod pests, particularly *Lygus* species (Hemiptera: Miridae) (Kaur et al. 2019).

Economically important species such as *Lygus lineolaris* migrate into crops during spring and summer, completing 2 generations per year and laying eggs on host plants (George et al. 2021). Using piercing-sucking mouthparts, these insects inflict damage during feeding through mechanical penetration and the injection of salivary enzymes, reducing yield, and seed quality (Williams et al. 2005). In faba beans grown for human consumption, even minimal damage can be economically significant: perforation exceeding the 1% limit for Canada Grade No. 1 results in crop downgrading and market losses (Canadian Grain Commission 2022). According to Cervantes et al. (2016), *Lygus* species employ their stylets and watery saliva to macerate plant tissues, forming pockets of disintegrated cells that are later flushed and ingested through abundant dilute saliva. Because *Lygus* lacks a salivary sheath during feeding, clear stylet tracks or sheath remnants are not left in plant tissues, making it difficult to determine the precise location of stylet insertion within the plant (Cervantes et al., 2016). As a result, correlating feeding waveforms from electrical penetration graph (EPG) recordings with histological observations remains challenging. Currently, the scouting protocol for *Lygus* species in faba beans in Canada recommends sampling with a standard 38-cm diameter sweep net on a sunny day when temperatures exceed 20 °C, and the canopy is dry, taking ten 180° sweeps at the field edge; however, no economic threshold has been established for *Lygus* species in faba beans in Canada.

This study investigates interactions between *Lygus* species and faba bean, focusing on factors associated with scarring damage on seeds. Specifically, the objectives were (i) to determine the relationship between the number of feeding *Lygus* individuals, feeding duration, and the extent of scarring damage in faba bean seeds and (ii) to identify the feeding duration necessary for an individual *Lygus* to cause visible damage. Based on these relationships, we identified the number of insects and duration of *Lygus* feeding activity that cause measurable economic losses in faba bean under controlled conditions.

## Materials and Methods

### Plant Materials

No-choice and EPG bioassays were conducted using the faba bean (*Vicia faba* L.) variety CDC 1310-5, obtained from the breeding program at the University of Saskatchewan Crop Development Centre. Plants were grown for 4 mo under controlled conditions at the University’s Controlled Growth Facility (Phytotron). The photoperiod was maintained at 18 h of light and 6 h of darkness with full-spectrum LED illumination. Daytime and nighttime temperatures were set at 21 °C and 18 °C, respectively, and relative humidity was set at 30%. Seeds were sown in 2-gallon (7.57 L) pots filled with Pro-Mix HP with Mycorise (Premier Tech Ltd., Rivière-du-Loup, QC). Pots were placed inside insect tents (75 × 75 × 115 cm; BugDorm, Taiwan). To prevent infestations by thrips and mites, the biological control agents *Orius laevigatus* and *Hypoaspis miles* (Biobest, Ontario, Canada) were released within each cage. Plants were irrigated every 4 days from emergence to flowering and fertilized weekly with a 20:20:20 (N: P: K) water-soluble fertilizer (Plant-Prod, Greater Toronto, Canada; 0.33 g/l). From flowering to seed maturity, watering frequency increased to every 2 days, and fertilizer was changed to a 15:30:15 formulation applied weekly.

### Insects

Two *Lygus* species were used in the no-choice bioassays, *L. lineolaris* (Palisot de Beauvois) and *L. elisus* (Van Duzee), whereas only *L. lineolaris* was employed for EPG experiments. Although *L. lineolaris* has been the more abundant species in faba bean production systems in recent years, *L. elisus* is also present in prairie agroecosystems and may co-occur within fields (Aguiar-Cordero and Prager 2025). However, because no-choice assays indicated that seed damage did not differ significantly between the 2 species, and given their comparable damage potential, *L. lineolaris* was selected as a representative species for detailed feeding behavior analysis using EPG. Colonies of both species were maintained on potato plants (*Solanum tuberosum*, var. Russet Burbank) inside growth chambers at the Phytotron. Potato plants served primarily as oviposition substrates and as a secondary food source. Although faba bean is a suitable feeding host, preliminary observations (Shahriar Jafari, unpublished data) indicated reduced reproductive performance when colonies were maintained exclusively on faba bean under controlled conditions. In contrast, reproduction and colony stability were more consistent on potato. Chambers were maintained under a 16 h light: 8 h dark photoperiod, with temperatures of 24 °C (day) and 21 °C (night), and 60% relative humidity. Potatoes were grown from seed pieces in 8-inch pots filled with Pro-Mix HP with Mycorise and enclosed in 60 × 60 × 60 cm BugDorm cages to facilitate oviposition. Organic romaine lettuce leaves were supplied as the primary food source, and plants were watered every 4 d.

### No-Choice Bioassay

**Fig. 1.**
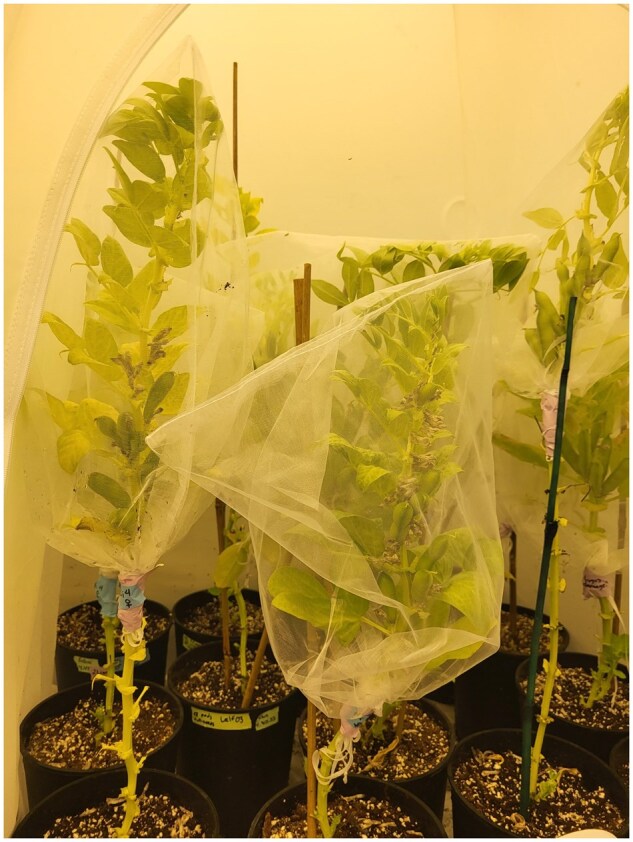
No-choice experiment using organza bags to confine *Lygus* individuals to plants.

The no-choice bioassay was designed to quantify the relationship between the number of feeding insects and the proportion of perforated faba bean seeds. When plants reached growth stage BBCH 72 (Biologische Bundesanstalt, Bundessortenamt and Chemical Industry scale), one adult *Lygus* was confined on a stem containing pods and flowers using a white organza bag (22 × 25 inches; ULINE, Edmonton, Canada) (Fig. 1). A preliminary trial using 4 insects per plant produced ∼3% seed damage within 24 h, exceeding the 1% economic threshold established for Grade No. 1 faba bean. Based on these findings, all subsequent assays used a single insect per plant to represent the minimum density capable of measurable damage. Feeding durations were set at 1, 3, 24, and 48 h. After each exposure period, insects were removed, and pods were examined for characteristic dark punctures indicative of feeding damage. Plants were then left to mature, and seed damage was quantified as (i) the proportion of perforated seeds and (ii) the percentage of total seed weight lost to perforation. In this experiment, each plant was considered a replicate; we used 10 plants with males and 10 plants with females for a total of 20 plants (replicates) with 10 per sex.

### Seed Evaluation

**Fig. 2.**
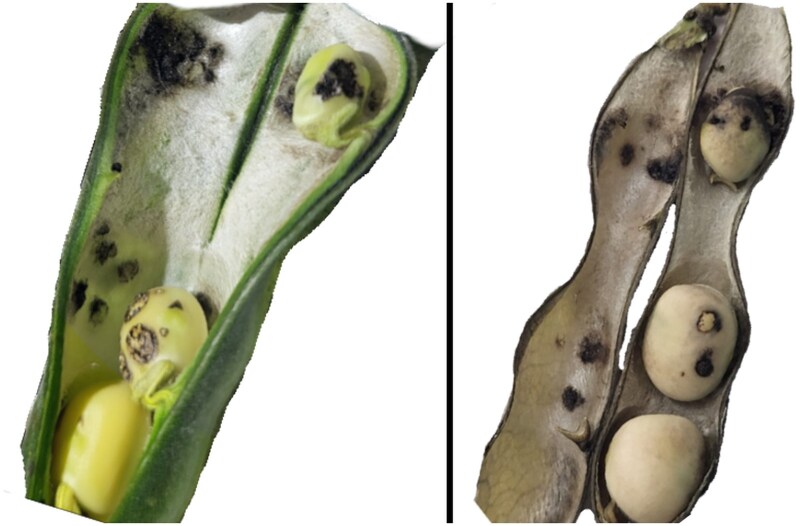
*Lygus lineolaris* damage to faba bean seeds from a no-choice laboratory bioassay. Shown at the immature (left) and dry (right) stages.

Seed damage was assessed only from pods enclosed within the organza bags. For each sample, all seeds were visually inspected and classified as either perforated (ie showing visible feeding punctures) or undamaged (ie without visible perforations) (Fig. 2). Perforated and undamaged seeds were weighed separately, and the percentage of perforated seeds and corresponding weight loss were calculated relative to the total seed sample. A non-infested control group was included, and no perforation damage was observed in any control replicate.

### EPG Bioassay

The EPG technique was used to determine the feeding duration required for a single *Lygus* to cause visible seed or pod damage. Experiments were conducted with nymphs instar 3 and adult *L. lineolaris*. Insects were food-deprived for 2 h, chilled on ice for 2 min inside a 1.5 ml microcentrifuge tube, and immobilized under a microscope using gentle suction. Each insect was then tethered with a 2 cm long, 0.0015-inch gold wire attached to the pronotum using water-based silver glue. Recordings were obtained using an 8-channel Giga-8dd Basic EPG system (DC amplifier, 10^7^ Ω) inside a Faraday cage. Six faba bean plants were positioned in plastic pots on Styrofoam bases within the Faraday cage to ensure electrical isolation. One pod per plant was selected for insect placement and connected to the EPG system, resulting in one insect per plant (6 insects per recording session). The number of plants per session was determined by the spatial and electrical constraints of the Faraday cage. Recordings lasted 6 h, as preliminary no-choice assays demonstrated that feeding sufficient to cause seed perforation occurred within 3 h. Extending the recording period to 6 h increased the likelihood of capturing sustained probing and salivation events while remaining within a practical timeframe for continuous monitoring under controlled conditions. After which insects were removed.

**Fig. 3.**
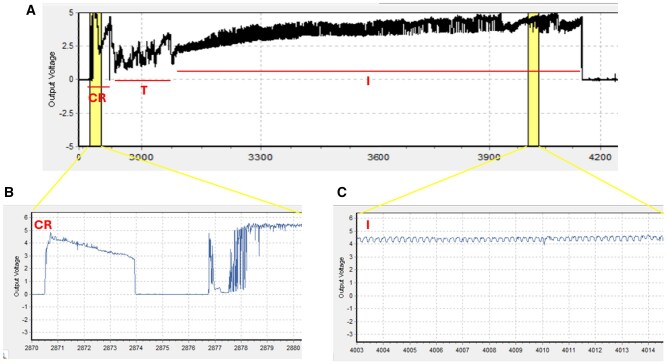
Representative feeding waveforms of *L. lineolaris* on faba bean pods during DC-EPG recordings. (A) Complete feeding sequence including CR, transition (T), and ingestion (I). (B) Detail of CR waveform. (C) Detail of I waveform.

Feeding behaviors were categorized into 6 waveform types following Cervantes et al. (2016): non-probing activities (standing, walking, antennation) and probing activities (CR, transition, ingestion). The 3 probing waveforms are particularly informative because they correspond to cell perforation and sap ingestion. The CR waveform began with a high-amplitude spike followed by irregular oscillations and typically transitioned into the T waveform, which preceded the ingestion (I) phase characterized by regular, high-frequency patterns (Fig. 3). Fifteen individuals, each of females, males, and 3rd instar nymphs (sex unknown) were analyzed.

Following EPG recording, pods and seeds were sectioned and stained to detect salivary residues using an adapted McBryde staining protocol (McBryde 1936; Prager 2011). Sliced pod and seed sections were immersed in McBryde’s stain (1:1 mixture of 95% ethanol and glacial acetic acid, supplemented with 5 ml of 0.02% aqueous fuchsin and 1 ml of acetic acid per 100 ml of solution) and incubated under a fume hood for 72 h. After staining, tissues were blotted dry and transferred to LGW clearing solution (lactic acid, glycerol, and distilled water, 1:1:1). Samples were then heated at 75 °C for 4 h in an aluminum tray. The cleared tissues were observed under a microscope to detect pink-stained salivary deposits in pod or seed tissue, indicating feeding sites. Waveforms were recorded using the EPG Stylet+d software (version 2023) and then analyzed using the EPG Stylet+a software (version 2023).

### Data Analysis

All statistical analyses were performed in R version 4.3.1 (R Core Team 2023) using a significance level of *P *< .05. Data were first examined for normality and homogeneity of variance, and assumptions were met. For the no-choice bioassay, 2-way ANOVA was used with feeding duration and *Lygus* species as explanatory variables and seed damage (percentage of perforated seeds or perforated seed weight) as the response variable. Post hoc pairwise comparisons were conducted using Tukey’s HSD test.

Linear regression was used to model the relationship between feeding duration and the weight of damaged seeds, with exposure time as the explanatory variable. For EPG data, the mean and standard deviation of feeding durations were calculated for each waveform category (CR, transition, ingestion) and for each sex or life stage (n = 15). Feeding behavior among males, females, and nymphs was compared using generalized linear mixed-effects models. Each insect represented an independent experimental unit (replicate). Individual insects were treated as random effects, while sex and waveform type were treated as fixed effects. Pearson’s correlation coefficients were computed between waveform duration and the proportion of samples with detectable salivary deposits. Finally, logistic regression was used to evaluate whether insect group, waveform type, or total number of feeding events significantly influenced the likelihood of detecting saliva within seed tissues.

## Results

### No-Choice Bioassay

Feeding by a single *Lygus* individual for 48 h resulted in an average seed weight loss of 12.54% and 13.22% perforated seeds. Seed damage increased consistently with feeding duration (Table 1), reaching ∼1% damage after only 3 h of exposure. Overall, feeding duration had a significant effect on seed damage (*F *= 6.242; *P *< 0.0001; df  =  3). Post hoc Tukey’s HSD tests indicated that 24 h of feeding produced significantly greater seed damage than 1-h exposure (*P *= 0.0392), and 48 h resulted in significantly more damage than both 1-h (*P *= 0.0039) and 3-h (*P *= 0.0087) exposures (Table 3). However, seed damage did not differ significantly between 24 h and 48-h treatments, suggesting that most measurable injury occurred within the first 24 h of sustained feeding.

**Table 1.** Percentage of seed and weight damage in faba bean caused by *Lygus* under different no-choice feeding durations

Number of *Lygus*	Time (h)	% of weight	% seeds
**4**	96	42.10%	43.70%
**1**	48	12.54%	13.22%
**1**	24	11.61%	13.29%
**1**	3	1.17%	1.20%
**1**	1	0.61%	0.81%

**Table 3.** Two-way ANOVA testing the effects of *Lygus* species, feeding duration, and their interaction on weight damage in faba bean

Factor	Degrees of freedom (df)	Sum of squares (Sum Sq)	Mean square (Mean Sq)	*F*-value	*P*-value
**Species**	1	1	0.9	0.005	0.941
**Hours**	3	2,944	981.2	6.242	**0.0008**
**Spp: Hours**	3	724	241.2	1.535	0.213
**Residuals**	72	11,318	157.2		

Significant *P*-values are shown in bold.

**Table 2.** Tukey’s HSD post hoc test comparing feeding duration and *Lygus* species effects on faba bean seed damage

Group	Comparison	Difference (Diff)	Lower CI (Lwr)	Upper CI (Upr)	Adjusted *P*-value (*P*_adj_)
**Species**	*L. lineolaris—L. elisus*	−0.207	−5.796	5.381	0.941
**Hours vs 1 h**	3 to 1	1.056	−9.371	11.484	0.993
	24 to 1	10.811	0.383	21.239	**0.039**
	48 to 1	14.031	3.604	24.459	**0.004**
**Hours vs 3 h**	24 to 3	9.755	−0.673	20.182	0.075
	48 to 3	12.975	2.547	23.402	**0.009**
**Hours vs 24 h**	48 to 24	3.220	−7.207	13.648	0.849

Significant *P*-values are shown in bold.

A strong positive relationship was observed between feeding duration and seed weight loss (*t *= 4.166; *P *< 0.0001; df = 78), indicating that longer feeding periods consistently led to greater seed damage. However, seed damage did not differ significantly between *L. lineolaris* and *L. elisus* (*F *= 0.005; *P *= 0.941), and no significant interaction between species and feeding duration was detected (*F *= 1.535; *P *= 0.213) (Table 2). These results suggest that the extent of seed damage is primarily influenced by feeding duration rather than *Lygus* species identity. Although *L. lineolaris* has been the more abundant species in faba bean in recent years, both *L. lineolaris* and *L. elisus* are present in production systems. In our no-choice assays, seed damage did not differ significantly between the 2 species, supporting the conclusion that feeding duration is a stronger determinant of injury than species identity under controlled conditions.

### EPG Bioassay

Building upon the results of the no-choice experiment, EPG recordings combined with McBryde staining were used to characterize the feeding behavior of *L. lineolaris* (Fig. 3). During the 6-h recording sessions, electrical waveforms were biphasic, most began with negative polarity, while some started positive, with waveform peaks oriented above and below the baseline.

**Fig. 4.**
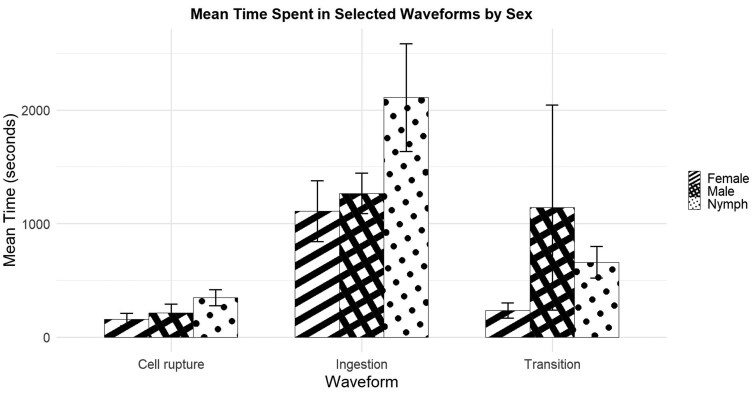
Mean total feeding time (seconds) spent by *L. lineolaris* females, males, and nymphs across major feeding behaviors (CR, T, I).

Feeding duration varied among waveform types (CR, Transition, and Ingestion) and insect groups (males, females, nymphs). Nymphs exhibited the longest mean duration for the CR waveform (348 ± 70.6 s), followed by males (215 ± 77.3 s) and females (157 ± 54.6 s). For the Transition phase, males spent the most time (1,143 ± 902 s), followed by nymphs (661 ± 138 s) and females (236 ± 67.8 s). The Ingestion phase was the longest across all groups, averaging 1,110 ± 269 s for females, 1,267 ± 179 s for males, and 2,110 ± 475 s for nymphs (Fig. 4).

There was no significant effect of insect group on feeding duration (ANOVA: *F*_2_,_225_ = 0.077; *P *= 0.926), whereas waveform type had a highly significant effect (*F*_5_,_225_ = 1,598.35; *P *< 0.0001), confirming that time allocation differed strongly among feeding activities but not among insect groups. Tukey HSD confirmed that the duration of each waveform type differed significantly, with ingestion being consistently longer than CR or transition events.

**Fig. 5.**
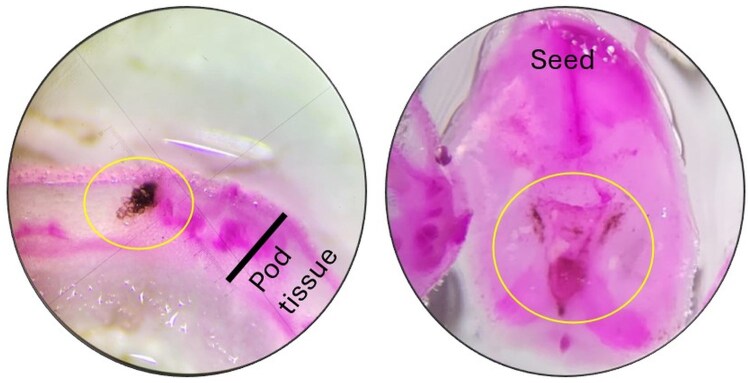
Examples of McBryde-stained pod and seed tissues from 6-h recordings showing saliva presence (yellow circles) in areas of *Lygus* feeding.

Analysis of McBryde-stained tissues showed no significant relationship between insect group, waveform type, total number of feeding events or the probability of detecting saliva in seeds. However, saliva was detected in only 5 replicates across all samples, resulting in a low frequency of positive observations and limited statistical power to detect meaningful associations (Table 4; Fig. 5). Odds ratios for these factors were close to 1, indicating little influence on saliva presence. Salivary residues were only detected in feeding from one female (*n* = 15) and from 4 males (*n* = 15), suggesting that saliva deposition occurred infrequently but was slightly more consistent among males. Nymphs exhibited no detectable salivary residues in seeds despite spending the most time feeding, potentially reflecting physiological or behavioral differences in feeding processes.

**Table 4.** Total number of feeding events, saliva presence, and total feeding time across *L. lineolaris* groups (female, male, nymph) and waveform types

Group	Waveform	Total event	Saliva in pods	Saliva in seeds	Total time (s)
**Female**	CR	106	2	1	1,885.3
	Ingestion	18	–	–	12,210.8
	Transition	25	–	–	2,356.19
**Male**	CR	147	2	4	3,108.61
	Ingestion	23	–	–	13,540.01
	Transition	32	–	–	12,818.33
**Nymph**	CR	261	0	0	4,877.05
	Ingestion	33	–	–	25,319.72
	Transition	75	–	–	7,926.58

## Discussion

The combined findings from the no-choice and EPG bioassays offer new insights into the feeding dynamics of *Lygus* species on faba bean, highlighting how feeding duration, life stage, and specific feeding aspects contribute to seed damage. These findings are not intended to support lower or automatic intervention thresholds, but rather to improve the biological understanding of how damage develops over time. By clarifying the duration of feeding required to cause measurable economic injury, management decisions can be better aligned with actual risk, potentially improving the timing of interventions and avoiding unnecessary insecticide applications when pest exposure is brief or transient. Together, these results provide a foundation for refining integrated pest management strategies aimed at reducing seed damage and maintaining faba bean quality.

In the no-choice bioassay, seed damage reached the critical 1% perforation threshold after only 3 h of feeding, which corresponds to the downgrade limit set by the Canadian Grain Commission. This indicates that even short periods of *Lygus* feeding can lead to economically significant quality loss. Similar results were reported by Turnock et al. (1995), who found that *Lygus*-induced seed collapse in canola ranged from 3% to 5%, reaching up to 20% in severely infested fields. The strong positive correlation between feeding duration and seed weight loss observed in this study reinforces the conclusion that longer feeding periods intensify the extent of damage. Therefore, the clear correlation between feeding duration and seed damage highlights that prolonged *Lygus* exposure increases the likelihood of economic losses in faba bean production.

Seed damage between *L. lineolaris* and *L. elisus* did not significantly differ, suggesting that both species exert comparable impacts on faba bean seed quality under controlled conditions. Although *L. lineolaris* has been the more abundant species in faba bean production systems in recent years, *L. elisus* is also present in prairie agroecosystems and may co-occur within fields (Aguiar-Cordero and Prager 2025). Given potential differences in species composition across landscapes and host availability, we initially evaluated whether species identity influenced damage severity. However, our results indicate that feeding duration, rather than species identity, was the primary driver of injury. This finding aligns with Armstrong et al. (2005), who reported that *L. elisus* and *L. hesperus* produced similar levels of injury on shared host plants.

EPG bioassays provided finer resolution of *L. lineolaris* feeding behavior on faba bean pods and seeds, revealing the electrical signatures associated with key feeding phases. Although the absence of salivary sheaths in most samples limited direct histological correlation, staining and waveform data support previous findings by Cervantes et al. (2017), showing that *Lygus* feeding induces structural alterations in host tissue. The observed waveforms corresponded to 3 primary feeding phases: CR, transition, and ingestion, each representing a critical step in the macerate-and-flush feeding process described by Backus et al. (2007). During the CR phase, the insect ruptures plant cells while injecting watery saliva that liquefies tissue; the T phase involves brief exploratory activity as the insect evaluates tissue acceptability; and the I phase represents sustained sap ingestion once cellular content has been solubilized (Cervantes et al. 2019).

Among the 3 *L. lineolaris* groups examined, nymphs spent the longest time feeding during both the CR and ingestion phases, suggesting greater potential for tissue disruption compared to adults. Fleury et al. (2006) reported similar developmental differences, finding that third-instar nymphs produced more punctures across plant stages than adults, despite similar overall feeding durations. Cervantes et al. (2019) likewise observed longer probing periods and shorter non-feeding intervals for nymphs on cotton, consistent with their intensive feeding activity. However, in the present study, nymphs did not deposit detectable saliva in seeds or pods despite prolonged feeding. This was not necessarily expected, as nymphs require continuous feeding for development. One possible explanation is that developmental differences between nymphs and adults influence saliva composition, injection rate, or volume, rather than overall feeding activity. It is also possible that nymphal saliva differs chemically in ways that reduce detectability using the staining method employed. Additionally, the handling and wiring required for EPG recordings may have affected smaller nymphs more strongly than adults, potentially altering normal feeding behavior. These possibilities highlight the need for further investigation into ontogenetic differences in feeding physiology and saliva composition among life stages.

Salivary deposits were found in a few samples from both male and female feeding, suggesting that adults may secrete saliva more consistently, perhaps due to more developed salivary systems or different feeding objectives. Cooper and Spurgeon (2010, 2011) demonstrated that feeding behavior and probing frequency in *Lygus* adults vary with sex, mating status, and reproductive condition. For instance, pre-reproductive adults spent more time feeding on cotton squares than reproductive ones, while mated males showed an increase in ingestion probes as accessory glands were replenished. These behavioral differences could partially explain why adult males exhibited more frequent saliva-positive samples in this study. Similarly, if salivation occurs during probing associated with oviposition, mated females could inflict greater injury to reproductive structures of host plants.

The presence of saliva in *Lygus* feeding sites has been linked to the release of pectolytic enzymes that degrade cell walls, contributing to tissue maceration and subsequent yield losses (Celorio-Mancera et al. 2008). Histological analyses by Cervantes et al. (2017) revealed clear, amorphous spaces in cotton tissue consistent with salivary enzymatic activity, which causes cytoplasmic degradation followed by ingestion of liquefied material. In this study, the limited detection of saliva, even among adults, may indicate that deposition depends strongly on feeding duration. This interpretation is consistent with the no-choice findings, where only 1% seed damage occurred after 3 h. Future research is needed to determine whether salivary enzyme activity differs across host plants and developmental stages and how these differences influence seed scarring in faba bean. Previous studies indicate that crop phenology strongly influences damage expression from Lygus feeding in different crops. Feeding during early reproductive stages, particularly on buds and flowers, can result in bud whitening and abscission, while feeding on developing pods and seeds may lead to pod abortion, seed shrinkage, chalk spot, and overall quality loss (Williams et al. 2005; Kaur et al. 2019; Beauzay et al. 2023).

Overall, these results highlight the importance of early *Lygus* management to prevent prolonged feeding and reduce the likelihood of economic damage. Understanding the behavioral and physiological mechanisms underlying feeding injury is valuable because it clarifies how rapidly economically meaningful damage can occur, thereby improving risk assessment and informing the development of more accurate economic thresholds under Canadian grading standards. Although farmers and crop consultants do not make management decisions based on feeding-time thresholds per se, quantifying the duration required to cause economic injury under controlled conditions provides a biological foundation for developing density-based economic thresholds under field conditions. Given that faba bean seeds reached the 1% economic damage threshold within just a few hours, current grading criteria and management thresholds may underestimate the potential for rapid injury. Recent field surveys conducted across Saskatchewan (Aguiar-Cordero and Prager 2025) demonstrate that *Lygus* species abundance and composition vary significantly among years and crop districts, with species-specific responses to climatic and geographic drivers. Such spatial and temporal variability suggests that localized colonization events or high-density populations could create high-risk scenarios in which economically significant injury develops quickly.

Because the thresholds established in this study were derived under controlled environmental conditions, field-based experiments will be necessary to validate these relationships under variable environmental conditions, plant phenology, and natural population densities. Incorporating these laboratory-based estimates into field trials could refine risk assessments and support the design of more effective integrated pest management strategies for faba bean and other pulse crops. Consequently, management strategies should focus on reducing feeding activity regardless of *Lygus* species, since damage potential appears to be consistent across the genus.
